# Frontal brain activity assessment during basic life support skill acquisition and transfer in serious game based learning environments

**DOI:** 10.1371/journal.pone.0354523

**Published:** 2026-07-31

**Authors:** Mehmet Emin Aksoy, Mert Deniz Polat, Patricia A. Shewokis, Dilek Kitapcioglu, Tuba Usseli, Atahan Agrali, Serhat Ilgaz Yöner, Kurtulus Izzetoglu

**Affiliations:** 1 Biomedical Device Technology Department, Vocational School, Acibadem Mehmet Ali Aydinlar University, Istanbul, Turkey; 2 CASE (Center of Advanced Simulation and Education), Acibadem Mehmet Ali Aydinlar University, Istanbul, Turkey; 3 School of Biomedical Engineering, Science and Health Systems, Drexel University, Philadelphia, Pennsylvania, United States of America; 4 College of Nursing and Health Professions, Health Sciences Department, Drexel University, Philadelphia, Pennsylvania, United States of America; 5 Department Medical Education, Medical Faculty, Acibadem Mehmet Ali Aydinlar University, Istanbul, Turkey; 6 Department of Anesthesia, Vocational School of Health Services, Acibadem Mehmet Ali Aydinlar University, Istanbul, Turkey; Politecnico di Milano, ITALY

## Abstract

Basic Life Support (BLS) is a crucial life-saving skill, and the effectiveness of different training methods for acquiring and transferring BLS knowledge and skills remains an important area of investigation. Traditional methods, such as lectures, may be less engaging and lack the practical application necessary for optimal skill transfer. New training modalities, including serious games on tablets and in virtual reality (VR) environments, offer interactive experiences, enable personalized feedback, and repetitive practice, potentially enhancing the learning process. However, the effectiveness of these newer training delivery methods on skill acquisition, transfer, and the underlying neurophysiological mechanisms needs further investigation. Hence, this study explores the effectiveness of various BLS training modalities, including traditional lecture-based instruction, tablet-based serious gaming, and VR-based serious gaming. A wearable functional near-infrared spectroscopy (fNIRS) sensory system is used to monitor prefrontal cortex (PFC) activity during training and subsequent hands-on testing. A total of 51 participants, who were randomly assigned to one of three training groups (lecture, tablet, VR), were analyzed. These participants underwent fNIRS monitoring during the training sessions and a final hands-on exam. Our findings revealed that while all three training modalities led to comparable hands-on exam performance, there were notable differences in neural activation patterns. VR-based training appeared to elicit neural responses indicative of more efficient use of cognitive resources during the hands-on test, suggesting its potential effectiveness as a practice mode for skill acquisition and transfer. These findings highlight the importance of considering both behavioral and neurophysiological metrics when assessing the efficacy of educational interventions.

## Introduction

By integrating elements such as competition, storytelling, and problem-solving into structured learning, serious games can provide more engaging, motivating, and memorable training experiences [1–3]. The use of serious gaming modules has expanded rapidly across many different industries like healthcare, aviation and military [4,5]. In healthcare, serious games are increasingly applied in surgical simulation, rehabilitation programs, mental health support, patient education, and emergency response training [6–9]. Serious gaming modules help learners improve clinical reasoning [10–12], teamwork, and procedural skills through immersive and realistic scenarios. Serious games offer safe, controlled, and cost-effective environments where users can practice complex tasks and decision-making without real-world risks. These modules provide students and trainees with flexibility regarding the time and location of their training [13]. Serious game-based learning tools have been widely used in medical students’ training to improve their knowledge and skills while their performances are closely tracked during the gaming [13,14]. The studies also reported that using serious gaming improves long-term retention of knowledge [13,15]. Further, personal computers (PC), tablet, and virtual reality (VR) based serious gaming modules are incorporated into learning tools for medical education [12,16–18]. However, the educational effectiveness and cognitive impact of these platforms require further evaluation using both conventional performance metrics and objective neurophysiological assessment methods, such as quantifying frontal activity during skill acquisition and transfer phases of the training, which may provide valuable insights into cognitive workload, neural efficiency, attention, and learning processes.

Hence, we investigated the effects of different serious game-based training and learning environments and evaluated their efficacy using performance scores and hemodynamic changes in the prefrontal cortex (PFC). Functional near-infrared spectroscopy (fNIRS) has been used to assess frontal brain activity changes. Unlike functional magnetic resonance imaging (fMRI) or electroencephalography (EEG), fNIRS is portable, low-cost, and tolerant to movement artifacts, allowing brain activity to be measured during real clinical settings such as surgical simulation or VR training [19–25].

Cognitive workload changes during learning and practice can be quantified with fNIRS by measuring real-time hemodynamic changes in response to brain activation. Several studies across different domains, ranging from aviation to healthcare, have used fNIRS to measure the PFC region of the brain, the area associated with the activations related to practice and skill acquisition [26] and in the assessment of the trainee’s cognitive workload [20,21,27–29]. The measurements acquired by fNIRS sensors during training are used as an assessment tool in medical simulation training in addition to the current scoring systems in different studies, as these integrated multi-assessments can reveal true workload changes during training [28,30–32].

In the present study, Adult Basic Life Support (BLS) training has been used as the training task, as the BLS course is one of the main courses for students in the anesthesia technician department of Acibadem Mehmet Ali Aydinlar University’s Vocational School in the second semester. Serious game-based BLS training used in this study includes Tablet PC and virtual reality-based training modules with different distraction levels.

The aim of this study is to reveal the learning outcomes of various BLS training methods, including traditional lecture-style instruction, tablet PC-based serious gaming, and virtual reality-based serious gaming by using optical brain imaging data in conjunction with participants’ behavioral data. The primary objective of this study is to determine an efficiency index by integrating optical brain imaging and behavioral data from participants to identify the most effective option for Basic Life Support training. While behavioral scores indicate that all groups learned the BLS protocol, these scores alone do not assess the neural activity associated with the task load. Hence, brain activity assessment is critical as it can serve as an index of the trainee’s neural “reserves,” a capacity that can be used to perform effectively under greater situational demands. Specifically, by utilizing brain imaging data, we aim to identify training methodologies that not only achieve high behavioral scores but also minimize neural activity in prefrontal areas, i.e., less cognitive workload. This is a critical factor that would help to ensure medical practitioners maintain mental capacity for real-world, high-stress clinical emergencies.

## Methods

Fifty-five subjects (47 female, 8 male) of Acibadem Mehmet Ali Aydinlar University’s Vocational School Anesthesia Technician Program volunteered to participate in this study. All participants had no prior knowledge about adult BLS guidelines. The mean age of the participants was 20.81 years (mean ± 0.52 years, SD). The exclusion criteria for the study included prior BLS training, a history of VR-induced motion sickness, and medical conditions such as vertigo attacks or the use of medications causing vertigo-like symptoms. Four volunteers were excluded from the study according to the exclusion criteria. Ethical approval for this study was obtained from the Ethical Committee of Acibadem Mehmet Ali Aydinlar University (registration number: 2021-21/36). All participants provided informed written consent, and the study was conducted in accordance with the Declaration of Helsinki. The recruitment period of the study was between 11th January 2022 and 9th February 2023. A pretest about the contents of the Adult BLS protocol was performed for all the participants in order to assess their initial knowledge and skill level about the adult BLS algorithm, which is a standardized, step-by-step sequence used to diagnose cardiac arrest, initiate CPR, and use an automated external defibrillator (AED). Adult BLS protocols are defined and periodically updated by organizations such as the American Heart Association (AHA) and the European Resuscitation Council (ERC). The content of the Adult BLS guide is summarized in Table 2.

**Table 1:** Comparison of lecture-based and serious gaming approaches for BLS training with fNIRS data collection.

Groups	Lecture Group	Serious Gaming Group	
		Tablet-Based Serious Gaming	VR-Based Serious Gaming
**Number of participants**	25	20	10
**Knowledge Domain**	Theoretical Lecture	Tablet PC based serious gaming module for BLS + fNIRS Data Collection	VR-based serious gaming module for BLS with a lower level of distraction + fNIRS Data Collection
**Psychomotor Skills**	Hands-on training with BLS manikin + with fNIRS Data Collection	Hands-on training with BLS manikin + with fNIRS Data Collection	Hands-on training with BLS manikin + with fNIRS Data Collection
**Assessment**	Evaluation by using the BLS manikin + with fNIRS Data Collection	Evaluation by using the BLS manikin + with fNIRS Data Collection	Evaluation by using the BLS manikin + with fNIRS Data Collection

**Table 2:** Scoring criteria of the tablet and VR based serious gaming modules and hands-on evaluation software.

*Criteria*	*Score*
*Check Safety*	5
*Check Consciousness*	9
*Head Tilt*	5
*Check Breathing*	6
*Call for Help*	5
*Get AED* ^*a*^	5
*Check Carotid Pulse* ^*c*^	0
*1*^*st*^ *CPRx30*^*b*^	4
*1*^*st*^ *Ventilation*	2
*2*^*nd*^ *CPRx30*^*b*^	4
*2*^*nd*^ *Ventilation*	2
*3*^*rd*^ *CPRx30*^*b*^	4
*3*^*rd*^ *Ventilation*	2
*4*^*th*^ *CPRx30*^*b*^	4
*4*^*th*^ *Ventilation*	2
*5*^*th*^ *CPRx30*^*b*^	4
*5*^*th*^ *Ventilation*	2
*2*^*nd*^ *Carotid Pulse*^*c*^	0
^*a*^ *AED Use Pad*	5
*AED*^*a*^ *On Off*	5
*Stand Clear*	5
*AED*^*a*^ *Shock*	5
*Final CPR* ^*b*^	15
*Check Rhythm*	0
*Total score*	100

^a^AED: automated external defibrillator.

^b^CPR: cardiopulmonary resuscitation.

^c^Healthcare Professionals only (not intended for use or evaluation in civilian populations).

The participants were randomized and then divided into three groups. The first group (Lecture Group), consisting of 25 participants, attended a theoretical lecture on the Adult BLS protocol. For this group, fNIRS data collection was not performed during the theoretical lecture; it was collected only subsequently during the hands-on practice and hands-on exam modules using the BLS manikin. The second group (Tablet PC Group), consisting of 20 participants, used a tablet PC based Adult BLS serious gaming module, whereas the third group, consisting of 10 participants, used a VR-based Adult BLS serious game, as seen in Table 1. The members of the second (Tablet PC Group) and third group (VR Group) were evaluated by the serious gaming module’s scoring system, and with the hands-on exam module following hands-on training with a BLS manikin.

The fNIRS experimental paradigm utilized a continuous, task-based block design. For the Tablet PC group, the protocol began with a 20-second resting baseline block, during which participants were instructed to remain still and relaxed. This was immediately followed by an active task block where participants completed the training mode. Following the training, a second 30-second resting baseline was recorded to allow hemodynamics to stabilize, followed immediately by the active exam mode block.

**Fig 1:**
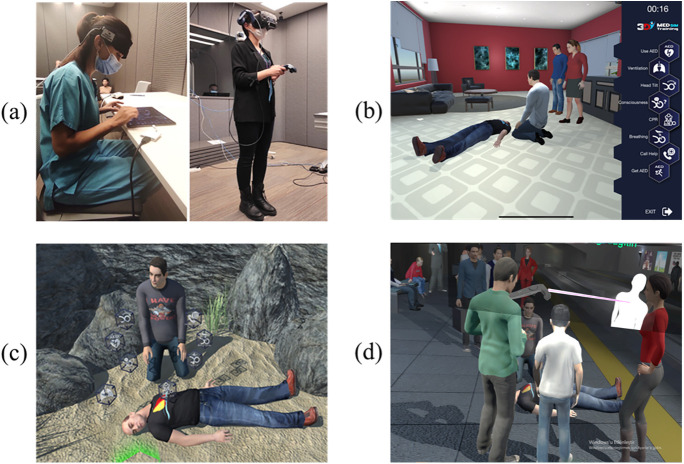
(a) Participants using tablet PC based and VR-based serious gaming modules. (b) Screen capture of a Tablet PC-based serious gaming module. (c) Screen capture of VR scenario with seaside environment with a low level of distraction. (d) Screen captures of a VR scenario in a subway environment with a higher level of distraction. (The screen captures in this figure originate from serious gaming modules developed with the scientific support of Acibadem University CASE. Therefore, there are no copyright issues associated with the use of these screen captures).

Similarly, the VR group used a continuous block design. An initial 20-second resting baseline was recorded prior to the active training mode block (seaside environment, low distraction). A subsequent 30-second resting baseline was recorded before the final active exam mode block (subway station scenario, higher distraction). fNIRS data collection was performed with the participants of the tablet PC based and VR-based serious gaming group during the training and exam modes (Fig 1a).

Screen captures of the tablet PC based and VR-based serious gaming modules are shown in Fig 1b–1d.

In the final phase of the study, all the participants of all groups had to take part in training with a BLS manikin in combination with fNIRS data collection. Following the training, all participants’ performances were evaluated by using a BLS manikin in combination with fNIRS data collection, as seen in Fig 1a. The details of the three groups are shown in Table 1.

Head-mounted displays (HTC Vive Pro 2®, Tauyan City, Taiwan) were used for this study, and the participants were informed about the potential risk of VR dizziness. Tablet PCs (Apple® iPad Pro 2, California, United States) were used for the tablet-based serious gaming module. Tablet PC and VR-based serious gaming modules (3DMedsim® GmbH, Bochum, Germany), hands-on evaluation software (3DMedsim® GmbH, Bochum, Germany), and a BLS manikin (CPR Lilly from 3B Scientific GmbH) were used for this study. The hands-on evaluation software was linked to the BLS manikins and could retrieve sensor data from the manikin via wireless network. As precise values of depth of chest compressions, compression frequencies and ventilations volume were obtained and scored by a dedicated software, it was possible to evaluate participants’ performances in parallel to their serious gaming performances [7]. Serious gaming scores and participants’ hands-on performances were stored on an individual basis by a dedicated learning management system (3DMedsim® GmbH, Bochum, Germany). The scoring criteria used by the serious gaming and hands-on evaluation module were based on the European Resuscitation Council 2021 Basic Life Support Guidelines [33]. The scoring criteria for the serious gaming and hands-on modules were identical, and the scoring criteria used by these modules can be seen in Table 2.

The serious gaming scores for the Tablet PC and VR group were collected using the modules’ embedded scoring systems. Performance scores were also provided by the hands-on module using the same criteria (Table 2). All these scores and fNIRS data were collected and evaluated for each participant.

**Fig 2:**
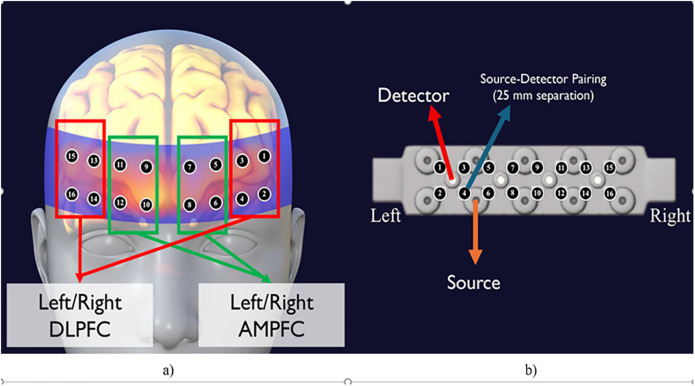
The continuous-wave functional near-infrared spectroscopy (fNIRS) system used in this study. (a) Sixteen measurement channels positioned over the prefrontal cortex, grouped into four regions of interest (ROIs): the left dorsolateral prefrontal cortex (LDLPFC), right dorsolateral prefrontal cortex (RDLPFC), left anterior medial prefrontal cortex (LAMPFC), and right anterior medial prefrontal cortex (RAMPFC). (b) The fNIRS sensor pad, consisting of four LED light sources and ten photodetectors, forms 16 measurement channels with a fixed source–detector separation of 25 mm.

A wireless fNIRS system (fNIR Devices, LLC, Potomac, MD, United States) was used in this study to monitor hemodynamic changes in participants’ prefrontal cortex (Fig 2).

The fNIRS data underwent preprocessing to address potential sources of interference, including instrument noise, motion artifacts, and physiological factors [34,35]. The preprocessing step employed a continuous wavelet-based motion artifact removal technique with an interquartile range (IQR) parameter of 1.5 [36], which corrects motion-induced spikes and baseline shifts without necessitating the complete exclusion of affected data segments [37,38], alongside a low-pass FIR filter with a cutoff frequency of 0.1 Hz to eliminate confounding signals such as cardiac and respiratory oscillations [35,36,39]. The filtered light intensity data were then processed using the Modified Beer-Lambert Law to obtain the relative oxygenated hemoglobin (HbO) and relative de-oxygenated hemoglobin (HbR) concentration measures [23]. The differential pathlength factor (DPF) used in this calculation was estimated from participant age and specific wavelengths, following the models established by Scholkmann and Wolf [40]. Given our continuous block design, relative concentration changes were calculated by block-averaging task periods relative to their baselines recorded immediately before task onset. Note that the HbO and HbR biomarkers were calculated relative to a baseline recorded during the 20-second and 30-second rest periods preceding each task. Consequently, the task-related brain activity reported in this study represents the relative change from this baseline period. For statistical analysis, block means were calculated by averaging the relative concentration changes over the entire duration of each task block. The regions of interest (ROIs) included the left and right hemispheres of the prefrontal cortex (PFC) and four quadrants: left dorsolateral prefrontal cortex (LDLPFC), left anterior medial prefrontal cortex (LAMPFC), right anterior medial prefrontal cortex (RAMPFC), and right dorsolateral prefrontal cortex (RDLPFC), as these regions are associated with brain activity during learning and training [20,26,41,42]. For each ROI, fNIRS signals were obtained by averaging the readings from predefined groups of spatially corresponding channels. These channel groupings are topographically mapped in Fig 2 and follow previously established protocols for this sensor array [20].

The sensor pad was positioned on the prefrontal cortex region of the participants, with the center of the array aligned to the Fpz landmark of the international 10–20 system and the lower edge resting just above the eyebrows (Fig 2). Measurements were obtained using near-infrared wavelengths of 730 nm and 850 nm, with a sampling rate of 5 Hz.

**Fig 3:**
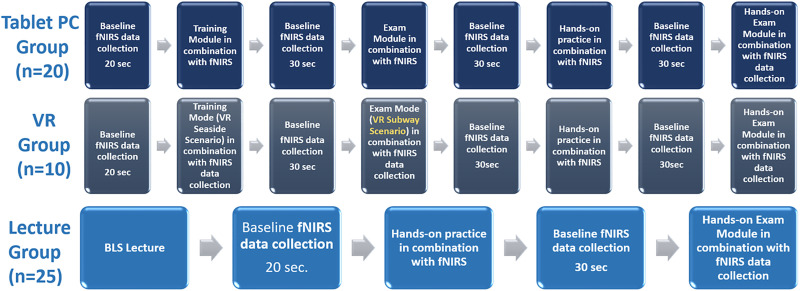
The study flow for the lecture group and the serious game-based groups.

The study flows for the lecture group and serious game-based groups are shown on Fig 3.

To further investigate trainee performance, the Relative Neural Efficiency (RNE) and Relative Neural Involvement (RNI) indices were calculated using standardized performance scores and hemodynamic parameters derived from fNIRS, including HbO, HbR, and OXY, in accordance with a previously described two-dimensional model [43–45]. In this model, P represents the standardized performance score obtained from the serious game Learning Management System (LMS) performance results, whereas M represents the standardized mental effort measure derived from fNIRS hemodynamic activity (HbO, HbR, or OXY values). Higher Relative Neural Efficiency (RNE) values suggest that trainees achieve superior task performance while using fewer neural resources, indicating a more efficient cognitive processing during task execution. In contrast, lower RNE values may reflect increased cognitive demand or less efficient allocation of neural resources to maintain performance. Higher Relative Neural Involvement (RNI) values indicate greater concurrent engagement of task performance and cortical activation, suggesting increased neural participation during learning and task execution [46–48]. Therefore, RNI may be interpreted as an indicator of the extent to which cognitive and neural resources are jointly mobilized during training. The RNI and RNE indices were calculated using Equations (1) and (2), respectively:


$$\begin{array}{c} RNI=\frac{\left(P+M\right)}{\sqrt{\text{2}}} \end{array}$$ (1)

$$\begin{array}{c} RNE=\frac{\left(P-M\right)}{\sqrt{\text{2}}} \end{array}$$ (2)

### Statistical methods

For the comparison of pretest scores across the three training groups, Welch’s one-way ANOVA was used due to unequal sample sizes across groups and its robustness to heterogeneity of variances [49] For the statistical testing for the comparison of Tablet and VR-Based BLS training modalities, linear mixed effects regression (LMER) modeling was used to accommodate the complex nested and crossed type structure and the missing data [50]. Linear mixed-effects regression (LMER) models, expressed using standard R formula notation in Eq. 3, were used to evaluate the main effects of Group (Tablet-based vs VR-based BLS Training groups) and Condition (Tablet/VR Training 1, Tablet/VR Training 2, Tablet/VR Exam). In this notation, the tilde (~) denotes ‘is modeled as a function of’ and the dependent variable (DV) represents the specific fNIRS-driven biomarker being analyzed iteratively (Left Hemisphere HbO, Left Hemisphere HbR, Right Hemisphere HbO, Right Hemisphere HbR, LDLPFC HbO, LDLPFC HbR, LAMPFC HbO, LAMPFC HbR, RAMPFC HbO, RAMPFC HbR, RDLPFC HbO, RDLPFC HbR) [51].


$$\begin{array}{c} \text{DV }\sim \;\text{1}\;+\text{ Group }+\text{ Condition }+\;\left(\text{1}|\text{Subject}\right) \end{array}$$ (3)

The statistical significance of the fixed effects was assessed using likelihood ratio tests, comparing a full effects model to one excluding the effect of interest. Effect sizes for the linear mixed effects models were calculated using marginal R-squared (R_m_^2^) to quantify the variance explained by fixed effects, and Cohen’s *f*^*2*^ to provide a standardized measure of local effect size where values of 0.02, 0.15, and 0.35 represent small, medium, and large effects, respectively [52,53]. Maximum likelihood estimation was used to conduct likelihood ratio tests, while restricted maximum likelihood was used to evaluate post hoc comparisons. When interaction terms were significant, planned comparisons (9 per fNIRS measures) compared blocks within groups (e.g., Training 1 vs Training 2 for Tablet-based group) and between groups for each task (e.g., Tablet-based vs VR-based group for Training 2). Model assumptions, including homogeneity of variance, normality of residuals, and random effects, were visually inspected using standard diagnostic plots. This included residual versus fitted value plots to assess homoscedasticity, and Quantile-Quantile (Q-Q) plots to evaluate the normality of residuals and random effects. If heteroscedasticity or non-normality was observed in model predictions, the response variables underwent log10 transformations. Post hoc analyses utilized the Satterthwaite approximation for degrees of freedom, adjusting p-values with false discovery rate (FDR) to counter Type I error inflation per dependent variable. Cohen’s d values were examined for post hoc effects, where d ≥ 0.8, 0.5, and 0.2 represented large, medium, and small effects, respectively [54].

For the statistical testing for the comparison between three groups (Lecture, Tablet, and VR-based groups), the Hands-on Exam was chosen to be a transfer task since it is a common task among all groups. The fNIRS-driven biomarkers were tested for their normality with the Shapiro-Wilk test for all groups, and revealed that a subset of such markers deviates significantly from normal distribution, suggesting nonparametric statistical testing [55]. Therefore, the Kruskal-Wallis H test is computed for testing between groups [56,57]. Effect sizes for Kruskal-Wallis tests were calculated using eta-squared (*η*^*2*^*H = (H – k + 1)/ (n – k)*), where H is the Kruskal-Wallis statistic, k is the number of groups, and n is the total sample size [58]. Values of approximately 0.01, 0.06, and 0.14 represent small, medium, and large effects, respectively [59]. For significant omnibus tests, Dunn’s test for pairwise post-hoc comparisons [56,57]. Adjustments using false discovery rate (FDR) were made on p-values in Dunn’s test to account for Type I error inflation per dependent variable. For all statistical analyses, the level of significance was set at α = 0.05. All statistical analyses were conducted in R (R Core Team, 2023) using *Dunn_test*, *Emmeans*, *Kruskal-Wallis.test*, *lme4, lmerTest*, and *rstatix* functions [60–63].

## Results

The pretest results revealed that the initial knowledge level score of the participants varied between 9/100 100/100, and the mean score was 62/100. A Welch’s one-way ANOVA was conducted to compare pretest scores among the three training groups. The analysis revealed no significant difference in pretest scores (*F*(2, 19.12) = 0.30, *p* = 0.741, η² = 0.03). The mean pretest scores were 64 (±22) for the lecture group, 60 (±16) for the tablet PC group, and 63 (±21) for the VR group as seen on Table 3. These results indicate that the three groups had comparable baseline knowledge prior to training.

**Table 3:** Descriptive statistics (mean ± standard deviation) for scores of groups.

Groups	Pretest Score /100	Serious Gaming Scores /100	Assessment with CPR Manikin Score /100
**Lecture**	64 ± 22		70 ± 9
**Tablet PC**	60 ± 16	76 ± 21	74 ± 8
**Virtual Reality**	63 ± 21	55 ± 5	73 ± 5

### Acquisition phase, fNIRS results

**Fig 4:**
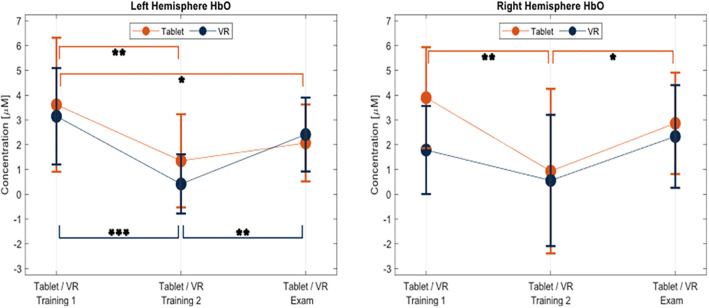
Left and right hemisphere relative concentration changes in oxygenated hemoglobin (HbO) block means during Tablet/VR Training 1, Tablet/VR Training 2, Tablet/VR Exam tasks for Tablet-based and VR-based groups. Note that the y-axis represents the relative change in concentration from the pre-task baseline. Markers denote group mean with standard deviation error bars (*: p < 0.05, **: p < 0.01, **: p < 0.001).

Linear Mixed Effects (LME) models were generated to investigate the effects of Group (Tablet-based vs VR-based BLS Training groups) and Condition (Tablet/VR Training 1, Tablet/VR Training 2, Tablet/VR Exam), in improving modeling fitness of fNIRS derived biomarkers, i.e., HbO and HbR block means from 2 PFC hemispheres (Left and Right) and 4 PFC quadrants (LDLPFC, LAMPFC, RAMPFC, RDLPFC). Fig 4 presents HbO and HbR block means from left and right hemisphere for the 3 tasks (Tablet/VR-Based Training 1, Tablet/VR-Based Training 2, Tablet/VR-Based Exam).

The effect of *Group + Condition* was significant for **Left hemisphere HbO** (*χ*^*2*^(2) =21.87, *p* < 0.001, *R*^*2*^*m* = 0.21, *f*^*2*^ = 0.27) and **Right hemisphere HbO** (*χ*^*2*^(2) = 15.46, *p* < 0.001, *R*^*2*^*m* = 0.18, *f*^*2*^ = 0.20). No significance was reported on HbR measurements.

Post hoc results from **the left hemisphere HbO** revealed significant differences for (i) Tablet-based group between Tablet-based training 1 and Tablet-based training 2 (*adj.p* = 0.003, *d* = 1.19), Tablet-based training 1 and Tablet-based Exam (*adj.p* = 0.026, *d* = 0.82), (ii) VR-based group between VR-based training 1 and VR-based training 2 (*adj.p* < 0.001, *d* = 2.84), VR-based training 2 and VR-based Exam (*adj.p* = 0.002, *d* = 2.10).

Post hoc results from **the right hemisphere HbO revealed significant differences for the Tablet-based group between Tablet-based training 1 and Tablet-based training 2 (adj p = 0.002, d = 1.22), Tablet-based training 2 and Tablet-based Exam (adj** *p* = 0.033, *d* = 0.78).

**Fig 5:**
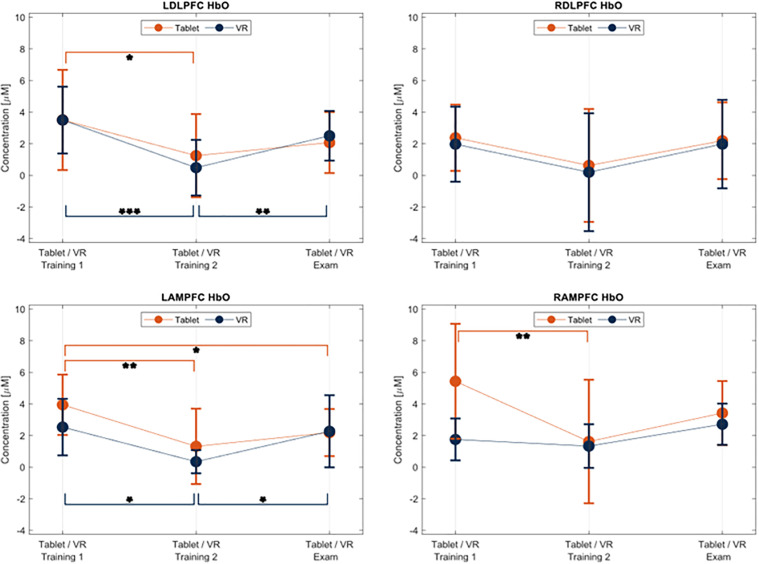
Left and right dorsolateral and anterior medial prefrontal cortex (LDLPFC, LAMPFC, RAMPFC, RDLPFC) relative concentration changes in oxygenated hemoglobin (HbO) block means during Tablet/VR Training 1, Tablet/VR Training 2, Tablet/VR Exam tasks for Tablet-based and VR-based groups. Note that the y-axis represents the relative change in concentration from the pre-task baseline. Markers denote group means with standard deviation error bars.

HbO and HbR block means from left and right dorsolateral and anterior medial prefrontal cortices (LDLPFC, LAMPFC, RAMPFC, RDLPFC) for the 3 tasks (Tablet/VR-Based Training 1, Tablet/VR-Based Training 2, Tablet/VR-Based Exam) are shown in Fig 5.

The effect of *Group + Condition* was significant for **LDLPFC HbO** (*χ*^*2*^(2) =17.22, *p* < 0.001, *R*^*2*^*m* = 0.16, *f*^*2*^ = 0.19), **LAMPFC HbO** (*χ*^*2*^(2) =22.04, *p* < 0.001, *R*^*2*^*m* = 0.23, *f*^*2*^ = 0.27), **RAMPFC HbO** (*χ*^*2*^(2) =12.30, *p* = 0.002, *R*^*2*^*m* = 0.18, *f*^*2*^ = 0.17) and **RDLPFC HbO** (*χ*^*2*^(2) = 7.28, *p* = 0.026, *R*^*2*^*m* = 0.08, *f*^*2*^ = 0.09).

Post hoc results from **LDLPFC HbO** revealed significant differences for (i) Tablet-based group between Tablet-based training 1 and Tablet-based training 2 (*adj.p* = 0.002, *d* = 1.22) (ii) VR-based group between VR-based training 1 and VR -based training 2 (*adj.p* < 0.001, *d* = 2.69), VR-based training 2 and VR -based Exam (*adj.p* = 0.006, *d* = 1.81).

Post hoc results from **LAMPFC HbO** revealed significant differences for (i) Tablet-based group between Tablet-based training 1 and Tablet-based training 2 (*adj.p* = 0.001, *d* = 1.63), Tablet-based training 1 and Tablet-based Exam (*adj.p* = 0.014, *d* = −1.11), (ii) VR-based group between VR-based training 1 and VR -based training 2 (*adj.p* = 0.016, *d* = 1.74), VR-based training 2 and VR -based Exam (*adj.p* = 0.017, *d* = 1.53).

Post hoc results from **RAMPFC HbO** revealed significant differences for Tablet-based group between Tablet-based training 1 and Tablet-based training 2 (*adj.p* = 0.002, *d* = 1.15).

### Transfer phase, behavioral results

Hands-on BLS exam is defined as the transfer phase, being the common task across all three groups (Lecture-based, Tablet-based, and VR-based groups). Acquisition of BLS exam scores has been detailed in previous sections. The Kruskal-Wallis test to examine the differences among groups has not revealed any significant differences in BLS scores (χ²(2) = 3.76, *p* = 0.152).

### Transfer phase, fNIRS results

fNIRS-derived biomarkers, i.e., HbO and HbR block means from 2 PFC hemispheres (Left and Right) and 4 PFC quadrants (LDLPFC, LAMPFC, RAMPFC, RDLPFC) for the Hands-on BLS exam were reported for the transfer phase. The Kruskal-Wallis test was conducted to assess the differences between the three groups (Lecture-based, Tablet-based, and VR-based groups), and Dunn’s test was utilized for pairwise comparisons post-hoc.

The Kruskal-Wallis test to examine the differences among groups has not revealed any significant differences in either hemisphere (Left HbO: χ²(2) = 5.53, *p* = 0.063, *η*^*2*^*H* = 0.08; Left HbR: χ²(2) = 4.64, *p* = 0.098, *η*^*2*^*H* = 0.06; Right HbO: χ²(2) = 3.13, *p* = 0.209, *η*^*2*^*H* = 0.03; Right HbR χ²(2) = 3.56, *p* = 0.169, *η*^*2*^*H* = 0.04). Post-hoc testing was not conducted since no significance was reported within multiple comparisons testing.

**Fig 6:**
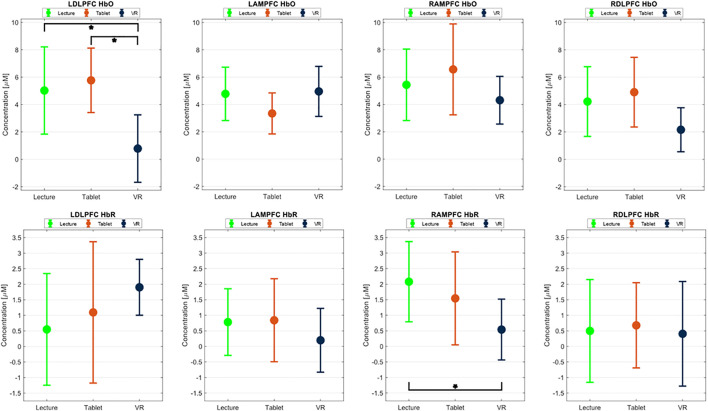
Left and right hemisphere oxygenated hemoglobin (HbO) and deoxygenated hemoglobin (HbR) block means during Hands-on Exam for Lecture-based (green), Tablet-based (orange), and VR-based (dark blue) groups. Markers denote group medians with median absolute deviation (MAD) error bars (*: p < 0.05).

HbO and HbR block means from left and right dorsolateral and anterior medial prefrontal cortices (LDLPFC, LAMPFC, RAMPFC, RDLPFC) for the Hands-on exam are shown in Fig 6.

The Kruskal-Wallis test to examine differences among groups has revealed significant differences in LDLPFC HbO (χ2(2) = 7.59, p = 0.022, η2H = 0.13) and RAMPFC HbR (χ2(2) = 6.60, *p* = 0.037, *η*^*2*^*H* = 0.11). Not significance was reported in other regions (LAMPFC HbO: *χ*^*2*^(2) = 0.41, *p* = 0.815, *η*^*2*^*H* < 0.01; RAMPFC HbO: *χ*^*2*^(2) = 1.87, *p* = 0.392, *η*^*2*^*H* < 0.01; RDLPFC HbO: *χ*^*2*^(2) = 4.82, *p* = 0.090, *η*^*2*^*H* = 0.07; LDLPFC HbR: *χ*^*2*^(2) = 4.14, *p* = 0.126, *η*^*2*^*H* = 0.05; LAMPFC HbR: *χ*^*2*^(2) = 0.14, *p* = 0.932, *η*^*2*^*H* < 0.01; RDLPFC HbR: *χ*^*2*^(2) = 0.64, *p* = 0.725, *η*^*2*^*H* < 0.01).

Therefore, post-hoc testing for pairwise comparisons via Dunn’s test was conducted at regions that had shown significance (LDLPFC HbO, RAMPFC HbR). Significant differences revealed for **LDLPFC HbO** between Lecture-based (*n* = 21) and VR-based (*n* = 8) group (*Z* = −2.19, *adj.p* = 0.043), Tablet-based (*n* = 17) and VR-based (*n* = 8) group (*Z* = −2.73, *adj.p* = 0.019) and for **RAMPFC HbR** Lecture-based (*n* = 21) and VR-based (*n* = 8) group (*Z* = −2.53, *adj.p* = 0.034)

### Transfer phase, neural efficiency, and involvement

**Fig 7:**
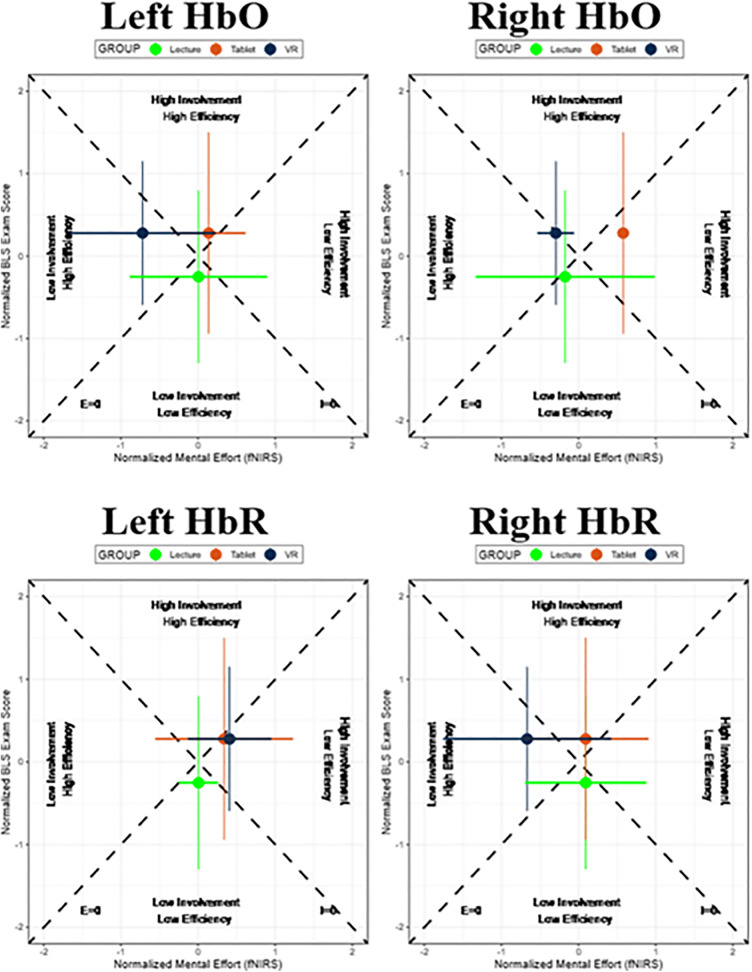
Relative neural efficiency (RNE) and relative neural involvement (RNI) metrics for Lecture-based, Tablet-based, and VR-based groups using Left and Right hemisphere oxygenated hemoglobin (HbO) and deoxygenated hemoglobin (HbR) fNIRS biomarkers. Markers denote group medians with median absolute deviation (MAD) error bars.

Relative neural efficiency (RNE) and relative neural involvement (RNI) metrics were calculated using Hands-on BLS exam scores and fNIRS-driven biomarkers, i.e., HbO and HbR block means from 2 PFC hemispheres (Left and Right) and 4 PFC quadrants (LDLPFC, LAMPFC, RAMPFC, RDLPFC). The Kruskal-Wallis test is conducted to assess the differences between the three groups (Lecture-based, Tablet-based, and VR-based groups), and Dunn’s test is utilized for pairwise comparisons post-hoc. Fig 7 presents RNE and RNI metrics for the three groups using Left and Right hemisphere HbO and HbR fNIRS biomarkers.

The Kruskal-Wallis test to examine the differences among groups has revealed significant differences in efficiency (RNE) for **Left HbO** (χ²(2) = 6.09 *p* = 0.048, *η*^*2*^*H* = 0.10) and **Left HbR** (χ²(2) = 6.15 *p* = 0.046, *η*^*2*^*H* = 0.10). No significant differences were reported for (i) efficiency (RNE) for Right HbO (χ²(2) = 3.55 *p* = 0.17, *η*^*2*^*H* = 0.04), Right HbR (χ²(2) = 1.36 *p* = 0.51, *η*^*2*^*H* < 0.01), (ii) involvement (RNI) for any biomarker (Left HbO χ²(2) = 1.91 *p* = 0.39, *η*^*2*^*H* < 0.01; Left HbR: χ²(2) = 0.22 *p* = 0.90, *η*^*2*^*H* < 0.01; Right HbO: χ²(2) = 1.60, *p* = 0.45, *η*^*2*^*H* < 0.01; Right HbR χ²(2) = 4.55 *p* = 0.10, *η*^*2*^*H* = 0.06).

Post-hoc testing was conducted on significant biomarkers (RNE-Left HbO, RNE-Left HbR) using Dunn’s test for pairwise comparisons. Significant differences revealed for **RNE-Left HbO** between Lecture-based (*n* = 21) and VR-based (*n* = 8) group (*Z* = 2.35, *adj.p* = 0.044), Tablet-based (*n* = 17) and VR-based (*n* = 8) group (*Z* = 2.18, *adj.p* = 0.044). No significant post hoc differences were found between any pairings for RNE-Left HbR.

**Fig 8:**
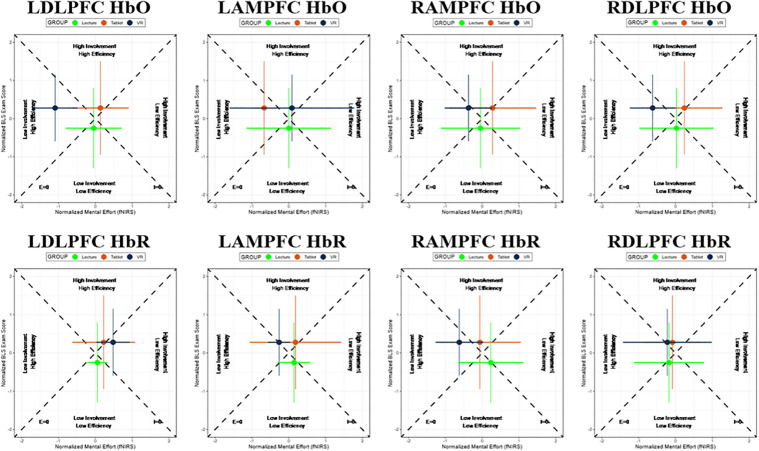
Relative neural efficiency (RNE) and relative neural involvement (RNI) metrics for Lecture-based, Tablet-based, and VR-based groups using oxygenated hemoglobin (HbO) and deoxygenated hemoglobin (HbR) block means from left and right dorsolateral and anterior medial prefrontal cortices (LDLPFC, LAMPFC,RAMPFC,RDLPFC) as fNIRS biomarkers. Markers denote group medians with median absolute deviation (MAD) error bars.

RNE and RNI metrics for the three groups using HbO and HbR block means from left and right dorsolateral and anterior medial prefrontal cortexes (LDLPFC, LAMPFC,RAMPFC,RDLPFC) for the Hands-on exam are shown in Fig 8.

The Kruskal-Wallis test to examine the differences among groups has revealed significant differences in (i) efficiency (RNE) for **LDLPFC HbO** (*χ²* = 6.99, *p* = 0.030, *η*^*2*^*H* = 0.12) and **RDLPFC HbO** (*χ²* = 7.62, *p* = 0.022, *η*^*2*^*H* = 0.14), (ii) involvement (RNI) for **RAMPFC HbR** (*χ²* = 9.50, *p* = 0.009, *η*^*2*^*H* = 0.17).

Post-hoc testing was conducted on significant biomarkers (RNE- LDLPFC HbO, RNE- RDLPFC HbO, RNI-RAMPFC HbR) using Dunn’s test for pairwise comparisons. Significant differences revealed for (i) **RNE- LDLPFC HbO** between Lecture-based (*n* = 21) and VR-based (*n* = 8) group (*Z* = 2.53, *adj.p* = 0.031), Tablet-based (*n* = 17) and VR-based (*n* = 8) group (*Z* = 2.32, *adj.p* = 0.031), (ii) **RNE- RDLPFC HbO** between Lecture-based (*n* = 21) and VR-based (*n* = 8) group (*Z* = 2.74, *adj.p* = 0.019), (iii) **RNI- RAMPFC HbR** between Lecture-based (*n* = 21) and VR-based (*n* = 8) group (*Z* = 3.08, *adj.p* = 0.006).

## Discussion

Our study evaluated the effectiveness of various training modalities for teaching BLS protocols to individuals without prior knowledge, underscoring the relevance of our findings for educational approaches to novice learners in the healthcare domain. By employing fNIRS to monitor hemodynamic changes in the prefrontal cortex (PFC) during both training and transfer phases, we gained insights into the cognitive workload underlying skill acquisition and transfer.

During the acquisition phase, our study revealed noteworthy trends in hemodynamic responses, particularly in left-hemisphere oxygenated hemoglobin (HbO) levels and in specific prefrontal cortex (PFC) regions (LDLPFC, LAMPFC). We observed significant decreases in HbO levels from Training 1 to Training 2 in both the tablet and VR-based groups, suggesting a training effect as participants became more familiar with the BLS protocols and interactive learning environments. This observation aligns with previous research indicating that skill acquisition is often associated with a reduction in cognitive workload as learners develop greater proficiency and automaticity [26].

Interestingly, we observed a subsequent increase in HbO levels from Training 2 to the tablet or VR-based exam in both groups. This rise in neural activation suggests heightened cognitive engagement and effort, possibly driven by the transition from a learning context to a performance evaluation setting. This finding emphasizes the dynamic nature of neural responses during skill acquisition, where even as overall cognitive workload decreases with practice, specific task demands or situational factors can elicit increased activation in relevant brain regions.

In the transfer phase, our examination of hands-on BLS exam results across all three groups did not reveal significant differences, suggesting comparable behavioral outcomes across the different training modalities, as shown in Fig 6. This finding suggests that all three training modalities (lecture-based, tablet-based, and VR-based) were equally effective in facilitating the transfer of BLS skills to a practical setting.

The findings of this study support the growing role of technology-enhanced learning environments in training critical clinical skills such as BLS. Simulation-based education aims not only to achieve adequate performance outcomes but also to optimize cognitive processes underlying learning, decision-making, and skill transfer. In particular, the higher neural efficiency observed following VR-based training indicates a potential advantage of immersive simulation in fostering more effective cognitive processing during complex, time-sensitive clinical tasks. This has important implications for curriculum design in medical education, where integrating advanced simulation modalities such as VR may enhance learners’ preparedness for real-world clinical environments.

As expertise tends to be associated with overall lower brain activity relative to novices, particularly in prefrontal areas [64]This study focused on the specific regions of interest (ROIs) within the prefrontal cortex (PFC) to explore brain activity changes during skill acquisition. The dorsolateral regions (DLPFC) are known to be engaged in executive functions, working memory, and active attention allocation [20,65]. Similarly, the anterior medial prefrontal cortex (AMPFC) is often associated with memory retrieval, action planning, and the regulation of physiological responses to demanding environments. The lower relative activation (higher efficiency) observed in the DLPFC and AMPFC could suggest that the VR group relied less on working memory and action planning during the transfer task. These neurophysiological changes suggest that VR training may offer an efficient mode of practice that helps develop expertise more quickly.

Our analysis of hemodynamic responses using fNIRS during the transfer phase revealed noteworthy patterns of neural activation. While we observed significant differences in LDLPFC HbO and RAMPFC HbR levels among the three groups, analysis of relative neural efficiency (RNE) metrics revealed significant differences across groups in left-hemisphere HbO, left-hemisphere HbR, LDLPFC HbO, and RDLPFC HbO. Figs 7 and 8 further reveal that subjects that went through the VR-based training showed higher neural efficiency than the two other modalities. These results highlight the multifaceted nature of skill transfer and the importance of considering both behavioral and neurophysiological metrics when assessing the efficacy of educational interventions.

While behavioral scores indicate that all groups successfully learned the BLS protocol, these scores alone do not assess the neural activity taxed for that performance [43]. The higher neural efficiency observed in the VR-based training group is significant because it indicates the development of “automaticity” (i.e., lower brain activity, particularly in prefrontal areas, with higher task performance) [26,44,45,64,66]. In a real-world application, such an assessment is critical as it can serve as an index of the trainee’s neural “reserves,” a capacity that can be used to perform effectively under greater situational demands. Therefore, the immersive and interactive nature of VR potentially allows for a more robust integration of knowledge, preparing students not just to pass an exam, but to perform reliably under the greater cognitive load.

Overall, our findings contribute to a further understanding of the neural mechanisms underlying skill acquisition and transfer in the context of real-world tasks, i.e., BLS training. The training effect is observed and characterized by a decrease in prefrontal activation with practice, as expected given the brain’s adaptability in response to learning. Furthermore, the observed variation in neural responses across task conditions highlights the complexity of the cognitive processes involved in skill development.

The sample sizes across training groups were unbalanced, and the relatively small number of participants in the VR-based group (n = 10) compared with the tablet (n = 20) and lecture (n = 25) groups may have limited statistical power and reduced the generalizability of the findings. This difference in cohort sizes arose from practical recruitment limitations. In addition, although the hands-on BLS exam was intended to assess skill transfer, all participants completed hands-on practice prior to the assessment. As a result, the outcomes likely reflect near transfer rather than far transfer of skills, which may have minimized observable behavioral differences between training modalities. While fNIRS provides a practical and ecologically valid method for monitoring brain activity during training, it is inherently sensitive to motion and systemic physiological influences, particularly in immersive environments such as VR. Although our continuous wavelet-based filtering approach is highly effective at salvaging hemodynamic signals without any exclusion of data points, extreme motions could still be found in the neural-activity-related signals. Moreover, interpretations of neural efficiency are based on established theoretical models and should be viewed as indirect; differences in neural activation may also reflect variations in cognitive strategies or task engagement rather than clear learning advantages. Finally, this study focused on immediate post-training outcomes, and the long-term retention of BLS skills across training modalities remains to be explored.

## Conclusion

Our study provides insights into the effectiveness of different training modalities for BLS skills. While all three modalities (i.e., lecture-based, tablet-based, and VR-based) led to comparable hands-on exam performance, there were notable differences in neural activation patterns observed through fNIRS. VR-based training appeared to help more efficient use of cognitive resources during the transfer task, possibly due to its immersive and interactive nature. These findings highlight the importance of considering both behavioral and neurophysiological metrics when assessing the efficacy of educational interventions. Future research can further explore the potential benefits of VR-based training in healthcare education and investigate the long-term retention of skills acquired through different modalities.

## Supporting information

S1 File All supporting documents relevant to this submission are provided as attachments. (DOCX)
