# Supplementary material for: Frontal brain activity assessment during basic life support skill acquisition and transfer in serious game based learning environments
Source: PLoS One. 2026 Jul 31;21(7):e0354523. doi: 10.1371/journal.pone.0354523 (PMC13426989; doi:10.1371/journal.pone.0354523)
Supplement: S1 File — (DOCX) [file pone.0354523.s001.docx]

**Descriptive Statistics and Statistical Tables**

**Descriptive Statistics – fNIRS Results (To be reported separately, or in appendix)**

**Acquisition Phase**

**Tablet Group**

| **Region** | **fNIRS**  **Biomarker** | **Tablet-based**  **Training 1** | | | **Tablet-based**  **Training2** | | | **Tablet-based**  **Exam** | | |
| --- | --- | --- | --- | --- | --- | --- | --- | --- | --- | --- |
|  |  | **n** | **mean** | **std.**  **dev.** | **n** | **mean** | **std.**  **dev.** | **n** | **mean** | **std.**  **dev.** |
| Left  Hemisphere | HbO | 19 | 3.614 | 2.704 | 19 | 1.35 | 1.881 | 18 | 2.07 | 1.553 |
|  | HbR | 19 | 0.221 | 2.35 | 19 | 0.608 | 2.127 | 18 | -0.228 | 2.071 |
| Right  Hemisphere | HbO | 19 | 3.9 | 2.039 | 20 | 0.934 | 3.324 | 18 | 2.864 | 2.048 |
|  | HbR | 19 | 0.72 | 2.26 | 20 | 1.258 | 2.239 | 18 | 0.347 | 1.227 |
| LDLPFC | HbO | 19 | 3.507 | 3.171 | 19 | 1.248 | 2.63 | 18 | 2.075 | 1.929 |
|  | HbR | 19 | 0.043 | 2.69 | 19 | 0.396 | 2.927 | 18 | -0.801 | 2.995 |
| LAMPFC | HbO | 13 | 3.948 | 1.911 | 14 | 1.318 | 2.387 | 14 | 2.191 | 1.493 |
|  | HbR | 13 | 0.502 | 1.053 | 14 | 0.812 | 1.492 | 14 | 0.923 | 2.739 |
| RAMPFC | HbO | 19 | 5.436 | 3.639 | 20 | 1.623 | 3.911 | 18 | 3.426 | 2.026 |
|  | HbR | 19 | 0.727 | 4.622 | 20 | 1.467 | 2.548 | 18 | 1.085 | 1.413 |
| RDLPFC | HbO | 18 | 2.38 | 2.097 | 17 | 0.628 | 3.572 | 17 | 2.191 | 2.427 |
|  | HbR | 18 | 0.36 | 2.269 | 17 | 0.281 | 2.462 | 17 | -0.429 | 1.839 |

**VR Group**

| **Region** | **fNIRS**  **Biomarker** | **VR-based**  **Training 1** | | | **VR-based**  **Training2** | | | **VR-based**  **Exam** | | |
| --- | --- | --- | --- | --- | --- | --- | --- | --- | --- | --- |
|  |  | **n** | **mean** | **std.**  **dev.** | **n** | **mean** | **std.**  **dev.** | **n** | **mean** | **std.**  **dev.** |
| Left  Hemisphere | HbO | 8 | 3.148 | 1.947 | 7 | 0.416 | 1.194 | 8 | 2.412 | 1.491 |
|  | HbR | 8 | -0.186 | 1.044 | 7 | 0.695 | 0.644 | 8 | -0.064 | 1.156 |
| Right  Hemisphere | HbO | 8 | 1.785 | 1.778 | 7 | 0.556 | 2.65 | 8 | 2.333 | 2.073 |
|  | HbR | 8 | 0.66 | 1.61 | 7 | -0.109 | 0.868 | 8 | -0.475 | 2.275 |
| LDLPFC | HbO | 8 | 3.5 | 2.114 | 7 | 0.487 | 1.759 | 8 | 2.506 | 1.572 |
|  | HbR | 8 | -0.541 | 2.121 | 7 | 0.789 | 1.827 | 8 | -0.04 | 1.764 |
| LAMPFC | HbO | 8 | 2.536 | 1.79 | 7 | 0.348 | 0.736 | 8 | 2.271 | 2.283 |
|  | HbR | 8 | 0.262 | 0.898 | 7 | 0.647 | 1.522 | 8 | -0.038 | 1.35 |
| RAMPFC | HbO | 8 | 1.756 | 1.325 | 7 | 1.336 | 1.381 | 8 | 2.719 | 1.305 |
|  | HbR | 8 | 0.759 | 1.045 | 7 | -0.433 | 1.477 | 8 | -0.209 | 1.344 |
| RDLPFC | HbO | 8 | 1.972 | 2.374 | 7 | 0.2 | 3.729 | 8 | 1.979 | 2.8 |
|  | HbR | 8 | 0.4 | 2.627 | 7 | -0.111 | 2.015 | 8 | -0.526 | 3.402 |

**Transfer Phase (Hands-on Exam)**

**Lecture Group**

| **Region** | **fNIRS**  **Biomarker** | **Lecture Group** | | | **Tablet Group** | | | **VR Group** | | |
| --- | --- | --- | --- | --- | --- | --- | --- | --- | --- | --- |
|  |  | **n** | **median** | **MAD** | **n** | **median** | **MAD** | **n** | **median** | **MAD** |
| Left  Hemisphere | HbO | 21 | 5.161 | 2.814 | 17 | 5.646 | 2.109 | 8 | 2.508 | 2.393 |
|  | HbR | 21 | 0.405 | 1.695 | 17 | 1.413 | 2.010 | 8 | 1.629 | 1.099 |
| Right  Hemisphere | HbO | 21 | 4.359 | 2.387 | 17 | 6.568 | 2.786 | 8 | 4.004 | 1.436 |
|  | HbR | 21 | 1.446 | 1.269 | 17 | 1.446 | 1.078 | 8 | 0.217 | 1.208 |
| LDLPFC | HbO | 21 | 5.028 | 3.187 | 17 | 5.772 | 2.352 | 8 | 0.787 | 2.462 |
|  | HbR | 21 | 0.548 | 1.795 | 17 | 1.095 | 2.272 | 8 | 1.902 | 0.898 |
| LAMPFC | HbO | 10 | 4.777 | 1.953 | 7 | 3.344 | 1.499 | 5 | 4.958 | 1.831 |
|  | HbR | 10 | 0.782 | 1.072 | 7 | 0.841 | 1.334 | 5 | 0.197 | 1.025 |
| RAMPFC | HbO | 21 | 5.439 | 2.615 | 17 | 6.568 | 3.327 | 8 | 4.312 | 1.745 |
|  | HbR | 21 | 2.079 | 1.290 | 17 | 1.543 | 1.495 | 8 | 0.540 | 0.978 |
| RDLPFC | HbO | 20 | 4.226 | 2.547 | 14 | 4.908 | 2.545 | 8 | 2.165 | 1.606 |
|  | HbR | 20 | 0.498 | 1.653 | 14 | 0.678 | 1.369 | 8 | 0.406 | 1.682 |

**Descriptive Statistics – Exam Scores**

| **Task** | **Lecture-based Group** | | | **Tablet-based Group** | | | **VR-based Group** | | |
| --- | --- | --- | --- | --- | --- | --- | --- | --- | --- |
|  | **n** | **median** | **MAD** | **n** | **median** | **MAD** | **n** | **median** | **MAD** |
| **Hands-on**  **Exam** | 25 | 69.40 | 6.618 | 20 | 74.40 | 6.800 | 8 | 73.38 | 4.875 |

**Statistical Tables – fNIRS scores acquisition phase**

| **Dependent Variable** | **Term** | **logLik** | **Chisq (*χ^2^*)** | **df** | **p-value** |
| --- | --- | --- | --- | --- | --- |
| HbO | | | | | |
| Left Hemisphere HBO | Group | -145.5410573 | 3.052261663 | 1 | 0.621 |
| Left Hemisphere HBO | Group + Condition | -122.9758136 | 45.13048733 | 6 | *****<0.0001 |
| Right Hemisphere HBO | Group | -146.3616127 | 4.114575908 | 1 | 0.180 |
| Right Hemisphere HBO | Group + Condition | -128.5351921 | 35.65284126 | 6 | *****0.0004 |
| LDLPFC HBO | Group | -145.7566828 | 2.621010626 | 1 | 0.965 |
| LDLPFC HBO | Group + Condition | -126.7079956 | 38.09737441 | 6 | *****0.0002 |
| LAMPFC HBO | Group | -106.3212242 | 0.181932021 | 1 | 0.365 |
| LAMPFC HBO | Group + Condition | -84.38429398 | 43.87386045 | 6 | *****<0.0001 |
| RAMPFC HBO | Group | -146.0848112 | 4.802677267 | 1 | 0.0551 |
| RAMPFC HBO | Group + Condition | -130.1398619 | 31.88989859 | 6 | *****0.0021 |
| RDLPFC HBO | Group | -136.1053611 | 1.18647845 | 1 | 0.706 |
| RDLPFC HBO | Group + Condition | -126.9847919 | 18.24113838 | 6 | *****0.0263 |
| HbR | | | | | |
| Left Hemisphere HBR | Group | -109.359 | 0.070 | 1 | 0.791 |
| Left Hemisphere HBR | Group + Condition | -108.078 | 2.563 | 2 | 0.278 |
| Right Hemisphere HBR | Group | -111.705 | 2.613 | 1 | 0.106 |
| Right Hemisphere HBR | Group + Condition | -110.469 | 2.472 | 2 | 0.291 |
| LDLPFC HBR | Group | -108.742 | 2.316 | 2 | 0.314 |
| LDLPFC HBR | Group + Condition | -109.900 | 0.005 | 1 | 0.944 |
| LAMPFC HBR | Group | -86.772 | 1.040 | 1 | 0.308 |
| LAMPFC HBR | Group + Condition | -86.031 | 1.482 | 2 | 0.477 |
| RAMPFC HBR | Group | -111.821 | 2.382 | 1 | 0.123 |
| RAMPFC HBR | Group + Condition | -111.762 | 0.118 | 2 | 0.943 |
| RDLPFC HBR | Group | -105.882 | 0.070 | 1 | 0.791 |
| RDLPFC HBR | Group + Condition | -104.991 | 1.782 | 2 | 0.410 |

**Statistical Tables – fNIRS scores transfer phase**

| **Dependent Variable** | **df** | **Chisq (*χ^2^*)** | **Adj. p-value** |
| --- | --- | --- | --- |
| Exam Scores | | | |
| SCORE | 2 | 3.76 | 0.1524 |
| HbO | | | |
| Left Hemisphere | 2 | 5.53 | 0.0630 |
| Right Hemisphere | 2 | 3.13 | 0.2088 |
| LDLPFC | 2 | 7.59 | *0.0225 |
| LAMPFC | 2 | 0.41 | 0.8148 |
| RAMPFC | 2 | 1.87 | 0.3924 |
| RDLPFC | 2 | 4.82 | 0.0897 |
| HbR | | | |
| Left Hemisphere | 2 | 4.64 | 0.0983 |
| Right Hemisphere | 2 | 3.56 | 0.1690 |
| LDLPFC | 2 | 4.14 | 0.1260 |
| LAMPFC | 2 | 0.14 | 0.9324 |
| RAMPFC | 2 | 6.60 | *0.0368 |
| RDLPFC | 2 | 0.64 | 0.7254 |
| Efficiency | | | |
| Left Hemisphere HbO | 2 | 6.09 | *0.0477 |
| Right Hemisphere HbO | 2 | 3.55 | 0.1696 |
| LDLPFC HbO | 2 | 6.99 | *0.0304 |
| LAMPFC HbO | 2 | 0.35 | 0.8383 |
| RAMPFC HbO | 2 | 1.46 | 0.4816 |
| RDLPFC HbO | 2 | 7.62 | *0.0222 |
| Left Hemisphere HbR | 2 | 6.15 | *0.0462 |
| Right Hemisphere HbR | 2 | 1.36 | 0.5058 |
| LDLPFC HbR | 2 | 0.02 | 0.9881 |
| LAMPFC HbR | 2 | 0.81 | 0.6663 |
| RAMPFC HbR | 2 | 1.27 | 0.5307 |
| RDLPFC HbR | 2 | 1.23 | 0.5394 |
| Involvement | | | |
| Left Hemisphere HbO | 2 | 1.91 | 0.3854 |
| Right Hemisphere HbO | 2 | 1.60 | 0.4493 |
| LDLPFC HbO | 2 | 2.53 | 0.2822 |
| LAMPFC HbO | 2 | 1.61 | 0.4466 |
| RAMPFC HbO | 2 | 3.83 | 0.1474 |
| RDLPFC HbO | 2 | 0.45 | 0.7967 |
| Left Hemisphere HbR | 2 | 0.22 | 0.8960 |
| Right Hemisphere HbR | 2 | 4.55 | 0.1026 |
| LDLPFC HbR | 2 | 5.22 | 0.0737 |
| LAMPFC HbR | 2 | 0.36 | 0.8354 |
| RAMPFC HbR | 2 | 9.50 | *0.0086 |
| RDLPFC HbR | 2 | 1.73 | 0.4208 |
